# Molecular Mechanism of Resveratrol and Its Therapeutic Potential on Female Infertility

**DOI:** 10.3390/ijms25073613

**Published:** 2024-03-23

**Authors:** Rebeka Podgrajsek, Helena Ban Frangez, Martin Stimpfel

**Affiliations:** 1 Department of Human Reproduction, Division of Obstetrics and Gynecology, University Medical Centre Ljubljana, 1000 Ljubljana, Slovenia; rebeka.podgrajsek@kclj.si (R.P.); helena.ban@kclj.si (H.B.F.); 2 Medical Faculty, University of Ljubljana, 1000 Ljubljana, Slovenia

**Keywords:** resveratrol, female infertility, endometriosis, polycystic ovary syndrome

## Abstract

Resveratrol is a polyphenol present in various plant sources. Studies have reported numerous potential health benefits of resveratrol, exhibiting anti-aging, anti-inflammatory, anti-microbial, and anti-carcinogenic activity. Due to the reported effects, resveratrol is also being tested in reproductive disorders, including female infertility. Numerous cellular, animal, and even human studies were performed with a focus on the effect of resveratrol on female infertility. In this review, we reviewed some of its molecular mechanisms of action and summarized animal and human studies regarding resveratrol and female infertility, with a focus on age-related infertility, polycystic ovary syndrome, and endometriosis.

## 1. Introduction

Infertility is a global healthcare problem, affecting around 8–12% of couples [1]. It is estimated that the female factor contributes to around 50% of couples’ infertility and can be classified as primary (no previously achieved pregnancy) or secondary (previously achieved pregnancies) infertility [1,2,3]. The leading cause of female infertility was reported to be due to polycystic ovarian syndrome (PCOS) [2,4]. Other causes include endometriosis, tubal obstruction due to sexually transmitted infections, uterine fibroids, and endocrine and uterine anatomical abnormalities [2,4]. However, in 10–17% of females, no cause is identified (idiopathic infertility) [5]. Despite that, some studies have reported that idiopathic infertility could be a consequence of imbalanced adaptive immunity [5,6] or genetic abnormalities [7].

Treatment options depend on the cause. Numerous animal and human studies have reported that some supplements could aid in ovulation, fertilization, embryo development, and improve infertility-related pathologies. Until now, beneficial effects were reported for dietary antioxidants (vitamin C and E) [8,9], L-arginine [10,11], multivitamin and mineral supplementation [12], inositol [13], calcium and vitamin D [14], melatonin [15], coenzyme Q10 [16,17], l-carnitine [18], selenium with vitamin E [19], α-lipoic acid and myoinositol [20], folate [21] or rather 5-methyltetrahydrofolate and vitamin B12 [22], and omega-3 fatty acid [23]. Of the potential supplements, polyphenols, including curcumin [24], quercetin [25], and rutin [26], were also associated with improved reproduction. One of the polyphenols is resveratrol, whose research has also greatly increased in recent years.

Numerous publications regarding the effect of resveratrol on infertility-related conditions have been published: for instance, there are studies exploring the role of resveratrol on endometriosis [27,28,29], PCOS [30,31], and infertility [32]. Nonetheless, as female infertility can be due to many causes, more studies are needed to overview the potential positive or negative effect of resveratrol on different female infertility-related conditions. In this article, we therefore review the role and potential protective effect of resveratrol on female fertility and describe the molecular mechanism behind its action.

## 2. What Is Resveratrol?

Resveratrol (IUPAC: 5-[(E)-2-(4-hydroxyphenyl)ethenyl]benzene-1,3-diol) [33] is a polyphenolic content present in grape and grape products, such as wine, Itadori tea, peanuts, blueberries, strawberries, pistachios, and mulberries [34,35,36]. It can be present in two isomeric forms; cist- and trans-, the latter being more prevalent [37]. In plants, it is produced as a protective component, where it has a role in defense against UV-related and other injuries, diseases, and pathogenic attacks [38,39].

Ever since its discovery in 1939 by Takaoka [40], the research on resveratrol has greatly increased. Currently, resveratrol has been reported as a protective factor in various chronic diseases like cancer, liver diseases, neurological diseases like Parkinson’s and Alzheimer’s disease, and cardiovascular diseases due to its antioxidant, anti-inflammatory, and immunomodulating activity [41]. Its pharmacological potential for the mentioned pathologies stems from affecting numerous molecules involved with different signaling pathways, such as p53, Bax/Bcl2, FOXO, PGE2, aromatase, NO, COX, STAT3, src, TNF, AMPK, SIRT1, and more yet to be explored [42]. Some of them are a direct target of resveratrol, including COX-1, COX-2, AKT-1, ATM, SIRT1, aromatase, etc. [43]. In the next section, we describe some frequently reported molecular targets of resveratrol action, studied; however, they are mainly targeted in non-reproductive cells.

## 3. Targets of Resveratrol and Its Molecular Mechanisms

### 3.1. Prostaglandin-Endoperoxide Synthase 1 and 2 (PTGS, PTGS2)

Resveratrol has been shown as a peroxidase-mediated reversible inhibitor of prostaglandin-endoperoxide synthase, better known as cyclooxygenase 1 (COX-1), and a low inhibitor of COX-2 [44]. COX-1 is continuously expressed in most of the tissues compared with COX-2, which is mostly expressed in parenchymal cells and occasionally in inflammatory, endothelial, muscle, and interstitial cells [45]. Prostaglandins formed by COX-1 are mainly involved with homeostasis [46]. Contrarily, COX-2 is associated with the production of prostaglandins, involved with the promotion of inflammation and mediation of pain [46]. Its expression is strongly induced by pathogenic stimuli like inflammatory mediators and cytokines (lipopolysaccharides) [47], radiation [48], growth factors [49], and oncogenes [50]. Later studies confirmed the inhibitory effect of resveratrol on COX-2 lipopolysaccharide or phorbol ester-induced expression [51,52,53]. In a study on mouse skin by Kundu et al., they proposed that resveratrol targets and blocks IκappaB degradation, which results in the suppression of nuclear factor-κappaB (NF-κappaB) activation, leading to COX-2 expression inhibition [51]. Cancerous cells frequently express higher concentrations of COX-2, its expression correlating with the clinical stage of cancer [54,55]. Colorectal and lung adenocarcinoma cancerous cells treated with resveratrol showed an inhibition of COX-2 expression [55,56], which resulted in inhibited malignant proliferation [55]. Some studies, however, observed some differences in the impact of resveratrol on COX-2 expression. In a study by Lin et al., they observed an increased expression of COX-2 in head and neck squamous cancerous cells after resveratrol treatment due to ERK1/2 activation. The ERK1/2 activation led to an upregulation of COX-2 and its accumulation in the nuclei, which leads to consequences in the phosphorylation of ser-15 of p53 and apoptosis of neck squamous cancerous cells [57]. 

The role of COX-2 is, however, not only limited to inflammation processes. In terms of reproduction, it was shown that mice deficient in COX-2 were infertile, displaying ovulation, fertilization, and implantation abnormalities [58,59]. Cox-2-/- mice presented abnormal gonadotropin-induced oocyte maturation and cumulus expansion, leading to ovulation irregularities [60]. However, in some reproductive disorders, the use of COX-2 inhibitors was beneficial as it led to reduced growth of the endometrial tissue in patients with endometriosis [61]. Compared with reproductive pathogenesis (endometrial cancer), resveratrol treatment (5.0 mg/kg) of normal endometrial cells did increase COX-2 expression and stimulation of endometrial proliferation, benefiting fertility and pregnancy [62], which suggests a cell type-specific effect of resveratrol and a potential cellular environmental role.

### 3.2. Tumor Protein p53 (TP53)

Another indirect target of resveratrol, however, is p53. *TP53* is a tumor suppressor gene, which is commonly mutated in different cancers [63]. The gene encodes the p53 protein, which controls different cellular processes, including cell cycle, apoptosis, and DNA repair [64]. During different cellular stresses, p53 can arrest the cell cycle, therefore allowing the cell to repair the injury and DNA damage. However, if the DNA damage is too severe, p53 can induce the event of apoptosis, therefore preventing the proliferation of genetically unstable cells and tumorigenesis [64]. p53 is important in reproduction. It is involved with embryo implantation regulation, as loss of p53 in female mice resulted in reduced implantation, pregnancy rate, and litter size [65]. In humans, genetic variants in p53 were also associated with reproductive outcomes in relation to implantation and pregnancy [66,67].

Resveratrol has been also associated with anti-cancer properties. Studies on cancer cell lines have shown the effect of resveratrol on the induction of apoptosis, transactivation of p53, and its expression [68,69,70]. It has been observed that resveratrol activates the mitogen-activated protein kinase (MAPK) and causes an increased value of p53 protein. MAPK induces the serine-15 phosphorylation of p53, which in turn leads to the binding of p53 to DNA and the apoptosis of cancerous cells (thyroid and prostate cells) [69,71]. Resveratrol was, besides the induction of p53 and Cdk inhibitor p21WAF1/CIP associated with the inhibition of cyclin D and cyclin-dependent kinase (Cdk) 4, therefore showing an anti-proliferative effect in the tested breast carcinoma cells [72]. In human cervical carcinoma cells, resveratrol has been also observed to upregulate the expression of B-cell lymphoma (Bcl)-2-associated X protein (BAX) and downregulate that of Bcl-2 and Bcl extra-large proteins, which have an anti-apoptotic activity [73]. It also upregulated the expression of caspase-3 and caspase-9, therefore activating the mitochondrial apoptotic pathway [73]. The activity of p53 is regulated by nicotinamide adenosine dinucleotide (NAD)-dependent deacetylase SIRT1 [74], involved with metabolism, environmental stresses, senescence, aging, cancer, and neurodegenerative diseases [75,76].

### 3.3. Sirtuin 1 (SIRT1)

In addition to the impact on p53, resveratrol is also an activator of SIRT1. SIRT1 was reported to regulate various molecular factors. This includes the negative regulation of p53, NF-κappaB, and mammalian target of rapamycin (mTOR) and the positive regulation of PPARG coactivator 1 alpha (PGC-1α) expression, promoting mitochondrial biogenesis and glucose metabolism [77,78]. SIRT1 has an important role in reproduction as mice with two null alleles were infertile [79]. SIRT1 was also reported to be involved with oocyte maturation, as a study on mouse oocytes reported a protective role of SIRT1 signaling against oxidative stress [80]. A negative role of SIRT1 was reported in endometriosis. SIRT1 was largely overexpressed in patients with endometriosis in comparison to healthy women, the expression correlating with the severity of endometriosis [81,82]. Mice developed with SIRT1 overexpression present subfertility and implantation failure [81]. Contrary results were observed in non-pathological mouse endometrium, where SIRT1 was shown as an important factor for decidualization and successful embryo implantation [83]. A study by Sirotkin et al. on porcine ovarian cells reported that resveratrol stimulates SIRT1 accumulation and apoptosis [84]. They also observed inhibition of cellular proliferation and progesterone release, and an increased release of estrogen and testosterone, strengthening the direct involvement of resveratrol on ovarian cellular functions.

On the molecular scale, SIRT1 is associated with p53. In a study on mouse embryonic stem cells by Suvorova et al., they reported that resveratrol activates p53, which is regulated by SIRT1 and AMP-activated protein kinase (AMPK) [85]. AMPK activation positively regulates p53 activity, while SIRT1 negatively regulates p53 activity. In the same study, this was also confirmed experimentally as SIRT1 inhibition led to greater p53 activity than resveratrol alone [85]. Activation of SIRT1 by resveratrol induces the deacetylation of p53, which decreases p53 activity and therefore enables cell survival, even under cellular stress [85,86]. The mechanism of resveratrol activity on two counteracting proteins (p53, SIRT1) was discussed by Brockmueller et al. [87]. They observed that treatment of colorectal cancer cells with a high concentration of resveratrol (>10 µM) led to p53 acetylation and the upregulation of p21, caspase-3, and BAX, leading to apoptosis. Contrarily, treatment with a lower concentration of resveratrol (<5 µM) led to an upregulation of SIRT1 and therefore a concentration-dependent downregulation of p53 and cell survival [87]. The results show a concentration-dependent effect of resveratrol, with high concentrations being pro-apoptotic and lower being anti-apoptotic.

### 3.4. Mammalian Target of Rapamycin (mTOR)

Resveratrol has also been observed to affect mTOR signaling, which affects various processes, promoting protein synthesis and growth and inhibiting autophagy [88]. Resveratrol was reported to inhibit PI3K [89], therefore inhibiting the activation of the phosphoinositide kinase-3 (PI3K)/protein kinase B (Akt)/mTOR pathway, as observed in smooth muscle cells [90], glioma [91], and melanoma cancer cells [92]. Resveratrol reduced the growth of melanoma cancer cells due to the inhibition of the PI3K/AKT/mTOR pathway [92]. A study on ovarian cancer cells observed the reduced phosphorylation Akt and mTOR [93]. Ovarian cancer cells treated with resveratrol induced changes in glucose utilization, which led to caspase-independent cellular death and autophagy [93]. Some studies also reported the role of the p38/MAPK signal pathway. Besides the inhibition of PI3K/Akt/mTOR, resveratrol was observed to activate the p38/MAPK pathway in non-small-cell lung cancer cells [94] and T-cell acute lymphoblastic leukemia cells [95], therefore promoting autophagy and apoptosis. As PI3K/Akt/mTOR is involved with cell growth, proliferation, and cell survival [96], its inhibition and the activation of the p38/MAPK pathway involved with inflammation and cellular death regulation [97] was observed to lead to autophagy and apoptosis in cancer cells. A new mechanism of mTOR inhibition by resveratrol was also proposed by Liu and Liu [98]. They reported that resveratrol inhibits mTOR by promoting the interaction between mTOR and its inhibitor DEPTOR.

Regarding the involvement of mTOR in reproductive processes, a study by Leconte et al. [99] observed a role of the Akt/mTOR signaling pathway in endometriosis. They showed that inhibition of Akt/mTOR signaling reduced endometriotic cell proliferation in vitro and in a mouse model [99]. Compared with endometriosis, stimulants of mTOR and PI3K in healthy mice were associated with the increased activation of primordial follicles, suggesting it as a potential new strategy for in vitro activation of primordial follicles in patients with a poor ovarian reserve [100]. In agreement with this, the inhibition of mTOR pathways in mice impairs oocyte meiosis and follicle formation [101], suggesting the role of mTOR in fertility [102].

### 3.5. Tumor Necrosis Factor (TNF)

The TNF family, including TNF-α, TNFβ, the Fas ligand (FasL), and TNF-related apoptosis-inducing ligand (TRAIL), present a group of cytokines involved with inflammation and apoptosis [103]. Regarding female infertility, patients with endometriosis with infertility presented higher concentrations of inflammatory markers IL-6, IL-10, IL-13, and TNF-α than controls [104].

Resveratrol has been observed to mediate an inhibitory effect on the TNF-induced activation of NF-κappaB, activator protein-1 (AP-1), and caspase-induced apoptosis [105]. The generation of reactive oxygen intermediates and lipid peroxidation influenced by it was therefore suppressed in myeloid, lymphoid, and epithelial cells [105], as was the expression of matrix metalloproteinase expression (MMP-9) in smooth muscle cells [106]. Resveratrol, due to the activation of SIRT1, also suppressed the fibroblastic overexpression of other pro-inflammatory molecular factors, like inducible nitric oxide synthase (iNOS), MMP-9, interleukin-1 beta (IL-1β), and IL-6 [107]. Resveratrol was additionally proven to suppress the Toll-like receptor-4 (TLR4)/NF-κB/TNF-α pathway in rats [108], involved with inflammation and the expression of nitric oxide in microglial cells [109].

### 3.6. Aromatase

Another molecule whose effect is mediated by resveratrol is aromatase. Aromatase is an enzyme that is involved with converting androgen to estrogens [110]. In infertility management, aromatase inhibition has been observed to be effective for the induction of ovulation (ovarian stimulation) in PCOS women with ovulatory problems [111,112]. Aromatase inhibitors, together with a gonadotropin suppressor, also showed potential by decreasing endometriosis-related chronic pelvic pain [113]. Besides infertility-related conditions, aromatase is also involved in carcinogenesis as it favors breast cancer proliferation, therefore presenting as an interesting molecular target for endocrine-responsive breast cancer [110]. Regarding resveratrol, studies observed that its administration was able to inhibit aromatase in breast cancer cells [114,115].

**Figure 1:**
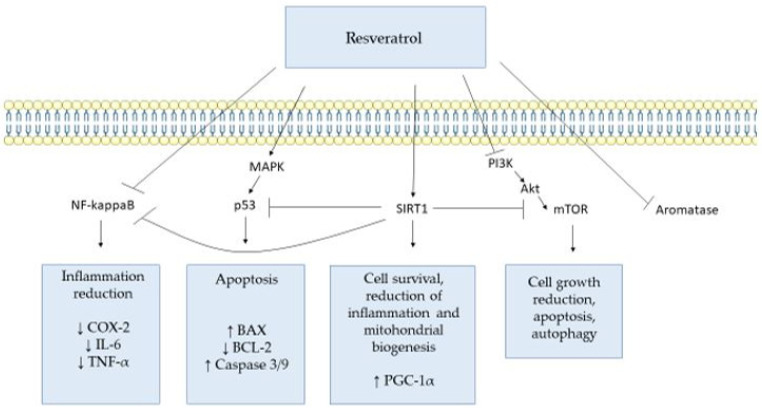
Overview of the main molecular targets of resveratrol on mainly non-reproductive cells, based on the reported studies. The arrows represent upregulation (↑) or downregulation (↓) of molecular factors. The figure was drawn using pictures from Servier Medical Art licensed under a Creative Commons Attribution 4.0 Unported License https://creativecommons.org/licenses/by/4.0/deed.en (accessed on 18 March 2024). COX-1/2: Cyclooxygenase 1/2; IL-6: interleukin 6; TNF-α: tumor necrosis factor alpha; BAX: BCL2-associated X, apoptosis regulator; BCL-2: BCL2 apoptosis regulator; PGC-1α: PPARG coactivator 1 alpha.

As reported, resveratrol targets many different molecular factors (Figure 1). Due to its numerous beneficial effects on non-reproductive cells, supplementation of resveratrol has been studied in various other diseases, including reproductive-related disorders and infertility, as discussed in the next section.

## 4. Resveratrol and Female Infertility

### 4.1. Resveratrol and Age-Associated Infertility

Age is an important factor in fertility, especially in females. In women, fertility begins to decrease at approximately 32 years and even more after the age of 37 years [116]. A study by Maheshwari et al. stated that women over 35 years old are also twice as likely to be affected by idiopathic infertility [117]. Age also affects the outcome of in vitro fertilization/embryo transfer (IVF-ET), whose success rate was observed to significantly decrease after the age of 34 years [118]. As studies have reported that the age-related fertility decline could be due to chronic inflammation, oxidative stress, and mitochondrial dysfunction [119,120], more research is being focused on studying those mechanisms.

#### 4.1.1. Animal Studies

Due to the reported anti-aging effect of resveratrol as reviewed by Zhou et al. [121], its long-term administration was tested on mice to explore its potential protective role in age-related infertility. Female mice were supplemented with ~7.0 mg/kg/day of resveratrol. Mice that were supplemented by resveratrol for 12 months presented a larger follicle pool compared with the control population. The gene expression profile, telomerase activity, and telomere length also differed from the control, proving that resveratrol promotes telomerase activity. Additionally, the quality and number of obtained oocytes were higher in the resveratrol-treated group. Interestingly, the dosage mattered, as a concentration of 0.1 µM benefits embryo development, whereas at a concentration of 0.5 µM, the benefits were not observed; higher concentrations of >1 µM showed an adverse effect in the form of cleavage arrest and embryo death [122]. In the study, they also showed that resveratrol increased the expression of Sirt1 in mice ovaries [122]. Other studies also reported SIRT1 as a positive regulator of telomere length, with its expression correlating with the telomere length [123,124]. Using chromatin immunoprecipitation assays, Palacios et al. reported the interaction of SIRT1 with telomere repeats in a mouse model [123]. 

Another study exploring the effect of resveratrol on the reproductive potential of aging mice was performed by Okamoto et al. [125]. Mice were fed with a diet (6 g daily), containing 0.04% (*w*/*w*) resveratrol for 0, 1, 12, and 22 weeks. The results showed that resveratrol treatment improved age-associated infertility and restored oocyte quality. Positive correlations were observed between resveratrol concentration and pregnancy and live pup rates. Resveratrol treatment also led to an increase in the mitochondrial membrane potential, ATP levels, and increased expression of ovarian SIRT1, SIRT3, SIRT4, SIRT5, and SIRT7. No impact, however, was observed on the estrous cycle, body weight, and copy number of mitochondrial DNA [125].

A study by Zhu et al. [126] reported the effect of resveratrol on ovarian aging in short-lived fish (*Nothobranchius guentheri*). They observed an increased expression of the proliferating cell nuclear antigen (PCNA), a marker of follicular growth [127] involved with cell cycle regulation and DNA repair [128]. Resveratrol also decreased the number of atretic follicles in the fish ovaries. It also increased the expression of SIRT1 and nuclear factor erythroid 2-related factor 2 (NRF2) [126], a regulator of oxidative stress and antioxidant response, promoting the expression of antioxidant enzymes, therefore protecting against ovarian aging [129]. Upon resveratrol supplementation, a reduction of inflammatory markers IL-1β, TNF-α, and IL-8, including the NF-κB transcription factor involved with the induction of cytokine expression [130], was observed [126]. Fish supplemented with resveratrol also presented with reduced levels of glucose-regulated protein 78 (GRP78), an endoplasmatic reticulum chaperone heat shock protein involved with unfolded protein response, a marker of chronic oxidative stress [131] and reduced levels of DNA damage-inducible transcript 3 (DDIT3), and another factor involved with the endoplasmatic reticulum unfolded protein stress response [132]. The knockdown of SIRT1 in HEK293T cells decreased NRF2 and increased inflammatory and endoplasmatic reticulum stress markers; this was, however, reversed by resveratrol supplementation, strengthening the involvement of SIRT1/NRF2 pathway in the reduction of ovarian aging [126].

#### 4.1.2. Human Studies

A study on humans was performed by Battaglia et al. [133]. In the retrospective analysis, aged women with poor ovarian reserve were treated with resveratrol and evaluated for the miRNA content in their follicular fluid. Six out of twelve women aged between 35–42 years old received a daily dose of 150 mg of resveratrol, 400 mg of folic acid, 25 ug of vitamin D, 2.5 ug of vitamin B12, and 1.4 mg of vitamin B6 for 3 months before the in vitro fertilization (IVF) cycle. After supplementation, they reported a significant increase in the mean number of fertilized good-quality oocytes. A total of 13 differentially expressed microRNAs were reported. A functional enrichment analysis of the differentially expressed microRNA showed that numerous targeted genes were involved with the oxidative stress response and oocyte maturation, strengthening the evidence of the beneficial effect of resveratrol on the improvement of oocyte quality [133]. 

Together with the mouse and fish models, all four studies reported beneficial effects of resveratrol on follicular pool or oocyte quality due to its beneficial effect on oxidative stress and inflammation, suggesting the benefits of resveratrol supplementation for oocyte quality and pregnancy outcomes in aged women. 

### 4.2. Resveratrol and Polycystic Ovary Syndrome

As reviewed by Azziz [134] and Lizneva et al. [135], PCOS is an endocrine–metabolic disorder affecting women of reproductive age. The diagnosis is based on symptoms, which include hyperandrogenism, polycystic ovarian morphology, and oligo-anovulation. Interestingly, individuals can still have the disease despite the absence of some typical symptoms. As PCOS is also a metabolic disorder, the majority of patients present chronic insulin resistance. PCOS patients also have an increased risk for type 2 diabetes mellitus, metabolic syndrome, non-alcoholic fatty liver, cardiovascular events, endometrial carcinoma, subfertility, anxiety, and depression [134,135]. In obese patients with PCOS, the first therapy includes lifestyle changes, like dietary changes, exercise, and potential dietary supplementation [134].

#### 4.2.1. Animal Studies

Numerous animal studies were performed regarding the effect of resveratrol on PCOS phenotype (Table 1) [136,137,138,139,140,141,142,143,144,145,146,147,148]. The majority of studies reported a beneficial effect of resveratrol on ovary morphology (improved follicular development) [137,138,141,142,143,144,145,146,148] body weight (fat) loss or shrinkage of adipocytes [136,138,141,143,145,148], and improved estrus cyclicity [136,143,145,146,148]. Additionally, improvement of the hormone profile or individual hormones [137,138,143,144,146,148], insulin sensibility [137,139,142,147,148], and oxidative stress were reported [137,138,139,141,142].

The effect of resveratrol on the inflammation markers and lipid profile was not as strong, as only three studies reported a decrease in Tnf-α [138,140,142] and one study reported a decrease in lipid markers (LDL, triglycerides) [142]. Despite the lack of evidence, the potential effect of resveratrol on inflammatory markers should not be rejected, as a recent study by Yuan et al. [149] reported the beneficial effects of resveratrol on oxidative stress and inflammation in granulosa cells from PCOS patients (downregulation of TNF-α, IL-1β, IL-6, and IL-8) due to the inhibitory effect on toll-like receptor 2 (TLR2) [149], which is involved with immune cell activation in relation to infections and autoimmunity (reviewed by Marks et al. [150]). 

Two studies [143,147] also observed an effect of resveratrol on SIRT2, a deacetylase involved with gluconeogenesis, the regulation of glycolysis enzymes, oxidative stress, and other metabolic processes [151]. Resveratrol was observed to restore the level of SIRT2, which was reduced in PCOS rats [143,147]. A study by Liang et al. [143] suggests that SIRT2 regulates the acetylation of glycolytic enzymes, as resveratrol supplementation also increased the expression of lactate dehydrogenase A (LDHA,) hexokinase 2 (HK2), and pyruvate kinase M1/2 (PKM2) [143,147,148], leading to an improvement of ovarian energy metabolism. Resveratrol was also observed to decrease factors associated with oxidative stress and fibrosis. One of them was transforming-growth-factor-beta(TGF-β) [141], involved with increased collagen disposition and fibrosis in ovaries, which leads to hyperandrogenism and ovulation irregularities [152]. Resveratrol also decreased the expression of other fibrotic factors, including β-catenin, α-smooth muscle actin (α-SMA), and connective tissue growth factor (CTGF) [141], including collagen IV and collagen IA1 [141]. In the same study, they reported an inhibitory effect of resveratrol on p66Shc expression. The p66Shc protein is involved with increased expression of fibrotic factors, which was, however, decreased upon resveratrol treatment. Resveratrol increased SIRT1, decreased oxidative stress, and inhibited phosphorylation of the protein, therefore suppressing the expression of other fibrotic factors and improving ovary morphology [141]. Upon resveratrol supplementation, a decrease in apoptosis was observed [148], which was molecularly strengthened by the increased expression of anti-apoptotic and decreased expression of pro-apoptotic factors [148]. 

Based on the current results from animal models, resveratrol presents various beneficial effects, especially on the ovary morphology (follicular development), due to the reduction of inflammation, oxidative stress, and improvement of the hormonal profile and ovarian metabolism. 

#### 4.2.2. Human Studies

Five randomized, double/triple-blind, placebo-controlled trial studies regarding resveratrol and PCOS were conducted (Table 2) [153,154,155,156,157]. All five studies reported the beneficial effect of resveratrol (800–1500 mg/day) on the PCOS phenotype. Their outcomes, however, vary due to the different study designs, or rather due to the exploration of different clinical parameters, as not all studies measured the same parameters. Banaszewska et al. reported that resveratrol treatment reduced ovarian and adrenal androgens and improved insulin sensibility [153]. As hyperandrogenism leads to excess hair, acne, and menstrual and ovulation abnormalities (reviewed by Ye et al. [158]), its decrease would improve the psychosocial and physiological well-being of those patients. Bahramrezaie et al. also observed reduced serum androgens (total testosterone) [154]. A recent study by Mansour et al., however, did not observe changes in the hormonal profile upon resveratrol treatment. Ovarian and adrenal androgens, sex hormone-binding globulin (SHBG) levels, free androgen index (FAI), lipid, and glycoinsulinemic profile were not significantly altered between groups [156]. Contradictory results were also observed regarding the potential of resveratrol to lower inflammatory markers. PCOS patients, as reviewed in Zhai and Pang, exhibit low-grade chronic systemic and ovarian inflammation [159]. Only one of two studies did, however, observe a beneficial effect of resveratrol on inflammation markers (reduction of IL-18, CRP, TNF-α, NF-κappaB), including markers of unfolding protein response (reduction of CHOP, GRP78, XBP1) [155]. Interestingly, one study [154] also observed a decreased expression of vascular endothelial growth factor (VEGF) and hypoxia-inducible factor 1 (HIF1). As reviewed by Di Pietro et al. [160], women with PCOS present with various abnormalities of ovarian angiogenesis. High androgen levels in PCOS women were associated with an increased activation of HIF1, an activator of VEGF, which stimulates endothelial proliferation, increasing the blood flow and additionally increasing the flow of growth factors and nutrients altering the follicular microenvironment [161]. Angiogenesis abnormalities may result in abnormal follicular development, presenting with anovulation and cyst formation [160]. Two studies explored the effect of resveratrol on ovary or fertility status. Bahramrezaie et al. reported a higher rate of high-quality oocytes and embryos [154], and Hashemi Taheri et al. reported an improvement in ovary morphology and follicular development upon resveratrol treatment [157]. Mansour et al. also observed better menstruation regularities after resveratrol treatment [156]. While animal studies (Table 1) reported improvement in ovarian morphology and function, only one study by Hashemi Taheri et al. was conducted to assess ovarian morphology by applying transvaginal ultrasound [157]. The results showed improvement in ovarian morphology, but due to the small sample size, more studies with larger participant numbers need to be conducted. Two additional randomized controlled trials on PCOS patients exploring the combinatory effect of resveratrol + myoinositol [162] and resveratrol + lipoic acid [163] were recently performed. Both studies observed beneficial effects on endocrine, stress, and metabolic parameters. 

Due to a limited number of obtained studies, the effect of resveratrol supplementation on humans is still understudied. Nevertheless, the obtained studies show great potential in the improvement of major PCOS irregularities, including the reduction of androgens, systemic inflammation, and fertility improvement due to potential cycle regulation, the improvement of ovary morphology, and the potential effect on oocyte and embryo quality.

### 4.3. Resveratrol and Endometriosis

Endometriosis, rather than being a disease, is a complex chronic inflammatory estrogen-dependent syndrome of women in adolescent or reproductive age (10–15%) in which the endometrial tissue can be observed outside the uterus; the complication, due to ectopic location, causes additional inflammation, fibrosis, and pain [164]. Patients present various symptoms, which can include chronic pelvic pain; painful periods (dysmenorrhea), intercourse (dyspareunia), defecation (dyschezia), and urination (dysuria); anxiety; and infertility [164,165]. Even though the majority of endometriotic lesions are located in the ovaries, fallopian tubes, and pelvic peritoneum, they can be also observed in other body locations, such as the gastrointestinal and urinary tract, adrenal glands, lungs, and nerves [166]. Clinical diagnosis is challenging due to the lack of non-invasive diagnostic tools and the large heterogeneity of symptoms, which are often overnormalized [167]. No curative treatment is currently available, and the therapy is focused on symptom reliance, which includes surgery, combined oral contraceptive pills, nonsteroidal anti-inflammatory drugs, selective estrogen receptor modulators, gestrinone, danazol, gonadotropin-releasing hormone agonists/antagonists, and aromatase inhibitors, as reviewed by Kalaitzopoulos et al. [168]. Other purposed alternative therapies consist of electrotherapy, acupuncture, dietary changes, and dietary supplementation [168].

#### 4.3.1. Animal Studies

Thirteen animal studies reporting the beneficial effect of resveratrol on endometriosis were observed (Table 3) [169,170,171,172,173,174,175,176,177,178,179,180,181]. The most reported effect of resveratrol was its ability to reduce the size and volume of endometriotic lesions [169,170,171,172,174,175,176,177,178,179,180,181]. Multiple studies also reported the effect of resveratrol on decreased angiogenesis, invasiveness, proliferation, and increased apoptosis [170,171,172,173,174,175,176,177,178,179,181]. 

The shrinkage of the endometriotic tissue and the confirmed increase in the apoptosis of the endometriotic lesions [170] could be explained based on the molecular mechanism of resveratrol action in the cell. Endometriosis tissue was reported to present a reduction in apoptosis, as confirmed by reduced p53 tissue staining and increased BCL-2 staining [182]. Upon resveratrol treatment, Zou et al. [181] observed an increased expression of p53 in endometriotic tissue. As mentioned in the section “Targets of resveratrol and its molecular mechanisms”, resveratrol has been reported to increase the expression and activity of p53, which is involved with the regulation of cell growth and apoptosis. In addition to the increase in p53, Chen et al. [179] also observed a decrease in BCL-2 expression upon resveratrol treatment. The anti-apoptotic BCL-2 protein is a member of the BCL-2 family of proteins, which includes pro- and anti-apoptotic molecules, with the BCL-2 being an anti-apoptotic protein. Increased expression of p53 induces pro-apoptotic proteins, leading to an inhibition of BCL-2 and activation of apoptotic effectors BAK and BAX, which leads to the release of cytochrome c, activation of caspases, and apoptosis (reviewed by Aubrey et al. [183]), which could explain the reduction in endometriotic lesion size. 

Matrix metalloproteinases are important for the progression and invasiveness of endometriosis. Its expression can be induced due to cytokines, oxidative stress, or environmental contaminants [184]. Resveratrol was observed to decrease the expression of MMP-2 and MMP-9 [176,179]. The observed reduction of MMP-2 and MMP-9 expression in the endometriotic tissue therefore suggests a positive role of resveratrol in the reduction of endometriosis invasiveness. Besides metalloproteinases, endometriotic tissue, similar to cancer, expresses higher levels of vascular endothelial growth factor (VEGF) involved with angiogenesis, which has an important role in disease progression [185]. Interestingly, supplementation of resveratrol did lead to a decrease in its expression [172,175,176,179]. Lower expression after resveratrol treatment was also observed for glucose transporters 1 and 3 (GLUT-1 and GLUT-3) and monocarboxylate transporters 1 and 4 (MCT-1 and MCT-4) [178], leading to the downregulation of glucose uptake, decreased glycolysis, and the production of lactate for vascular signaling [178]. 

Additionally, resveratrol was also reported to increase oxidative stress [174,181] in endometriotic cells, reduce inflammatory markers [172,175,176,180], and change some lipid markers [179]. As endometriosis also has an inflammation component, it was interesting to observe that resveratrol did reduce the expression of various inflammatory markers, including monocyte chemotactic protein-1 MCP-1 [172,175], which was observed to be overly expressed in endometriosis patients with worse prognosis [186], as well as the expression of INF-γ, interleukin-6 (IL-6) TNF-α, and interleukin-8 (IL-8) [176,180]. Interestingly, resveratrol was also able to induce the expression of peroxisome proliferator-activated receptor gamma (PPARγ) [180], whose agonists are emerging as a potential therapy in endometriosis due to its effect on the reduction of inflammation, adhesion, angiogenesis, and induction of apoptosis [187]. Based on the current results from animal models, resveratrol presents various beneficial effects on endometriosis, mainly due to its effect on reduced inflammation, invasiveness, proliferation, and increased apoptosis, leading to its size reduction. 

#### 4.3.2. Cell Models

The effect of resveratrol was also studied on human endometrial cells. Treatment of human endometrial stromal cells (ESCs) with resveratrol (10–30 μM) led to a concentration-dependent decrease (up to 78%) in their invasiveness [169]. Resveratrol was also observed to decrease vascular density and proliferation and increase apoptosis in a primary culture of human endometrial epithelial cells (EECs) [170]. Treatment of primary cultures of ESCs with resveratrol also resulted in decreased cholesterol biosynthesis, 3-hydroxy-3-methylglutaryl-coenzyme A reductase (HMGCR) activity, and its mRNA expression [188]. Resveratrol has also been reported to decrease inflammation in endometrial stromal cells due to the suppression of TNF-α-associated IL-8 expression [189]. In their other study, Taguchi et al. reported that resveratrol alone did not induce, but rather enhanced apoptosis in ESCs due to the suppression of survivin expression and regulation of TRAIL-induced apoptosis [190]. It also reduces the expression of IGF-1 and hepatocyte growth factor (HGF) protein in ESCs [191]. In a study by Khazaei et al. [192], they reported that resveratrol decreased the growth and angiogenesis of human endometriotic tissue, which was dose-dependent. They also observed a decrease in NO levels and an increase in apoptotic factors (Bax, p53, caspase-3) and SIRT1 [192]. Higher concentrations of resveratrol (200 μM) were observed to reduce eutopic endometrial stromal cells by 50%, while no such effect was observed at lower concentrations [193]. The expression of BCL-2 was increased in non-endometriotic controls and eutopic ESCs, while no significant differences were observed in the ectopic ESCs [193].

Resveratrol has been observed to decrease the gene and protein expression of MCP-1, IL-6, and IL-8 in eutopic and ectopic ESC and the regulated upon activation, normally T-expressed, and presumably secreted (RANTES) in ectopic ESCs [194]. It also decreased the expression of MMP-9, VEGT, and TGF-β in ESCs [195]. Chen et al. [179] also reported that resveratrol reduced the proliferation and invasiveness and increased the apoptosis of human ectopic ESCs. It also altered the lipid profile in human ectopic ESCs and induced the activation of PPARα [179]. Madanes et al. reported the effect of resveratrol on primary ESCs, immortalized ESCs (St-T1b), and EECs (12Z) [196]. Resveratrol did decrease the cell viability and migration and increased the apoptosis in the two cell lines. Upon treatment, all three cell populations showed decreased VEGF, MMP-2/TIMP-1, and Ang-1 expression. Resveratrol also affected stem cell phenotype, increasing the expression (100 µM) of Notch-1, KLF-4, TERT, and SOX-2 in all three groups [196]. Resveratrol and its analogs also preserve a toxic effect on endometriotic epithelial cells (12Z), such as inhibition of adhesion and cell growth, induction of apoptosis, and loss of cell content. It also induced dose-dependent DNA fragmentation and caspase-3 and caspase-7 activity. ESCs were observed to be more resistant to its effect at higher concentrations (100, 200, 400 µM) compared with 12Z cells [197]. Another study by Zou et al. explored the effect of resveratrol on ectopic ESCs, where they showed that resveratrol promoted oxidative stress and inhibited the proliferation and migration of ESCs due to ferroptosis [181], a non-apoptotic, iron-dependent cell death. 

#### 4.3.3. Human Studies

Four human studies exploring the effect of resveratrol were conducted (Table 4) [198,199,200,201], three of them being randomized, double-blind, placebo-controlled trials [199,200,201]. The results of an experimental study [198] reported the pain relief property of resveratrol. As inhibition of COX-2 and aromatase were reported to be beneficial for reducing endometriosis-related pain [113] and the reduction of its growth [61], the confirmed decrease in COX-2 and aromatase upon resveratrol treatment and reduction of pain in endometriosis patients with oral contraceptives is promising. Unfortunately, the pain relief properties of resveratrol were not confirmed in the randomized, double-blind, placebo-controlled trial [199]. However, there was a statistically significantly decreased expression of VEGF, a gene involved with angiogenesis, TNF-α, and metalloproteinases MMP-2 and MMP-9, involved with extracellular matrix breakdown [200,201]. This result suggests a potential anti-inflammatory and anti-angiogenesis role of resveratrol on endometriotic tissue and a protective role in the progression and invasion of endometriosis.

### 4.4. Resveratrol and Female Infertility Due to Other Causes

Besides age and reproductive disorders (PCOS and endometriosis), other factors can impact fertility. One of them is obesity, which is associated with an increased risk of infertility [202]. Abdominal obesity can also induce systemic oxidative stress independently of PCOS [203]. Resveratrol has been observed to have a protective impact on human granulosa cells due to the reduction of oxidative stress markers, cell death, and enhanced mitosis [204]. Resveratrol was also observed to support the growth of human ovarian follicles in a cell culture model [205].

A study by Bódis et al. [206] explored the role of SIRT1, SIRT6, and resveratrol in women undergoing in vitro fertilization, where the authors confirmed roles only for SIRT1 and SIRT6 and not resveratrol in human reproduction. In this study, however, resveratrol was not supplemented like in other studies; therefore, very low concentrations of resveratrol were measured, which could be one of the reasons for the reported failure to find an association of resveratrol with human reproduction [206]. Resveratrol treatment has shown a beneficial impact on the chemotherapeutic drug doxorubicin-induced meiotic failure. Resveratrol treatment improved mouse oocyte quality due to spindle assembly and chromosome rearrangement restoration. It also reduced oocyte apoptosis and ROS levels, proving the protective role of resveratrol against doxorubicin damage [207].

Resveratrol has also been observed to improve ovarian function in infertile women, as lower concentrations of resveratrol (1 μM and 10 μM) decreased the apoptosis of human granulosa cells. This was confirmed on the molecular level as Bcl-2 was increased and transforming growth factor-β (TGF-β), Bax, and caspase 9 were decreased upon resveratrol treatment [208].

#### 4.4.1. Animal Studies

Nine animal studies regarding resveratrol supplementation and female infertility due to other causes were retrieved (Table 5) [209,210,211,212,213,214,215,216,217]. Four studies explored the potential protective effect of resveratrol on chemotherapeutic-induced ovary toxicity [212,214,215,217], as well as one each for fungicide [211] and chromium-induced toxicity [210]. All studies reported a protective impact of resveratrol on ovary toxicity, including a reduction in apoptosis, molecularly strengthened by the reduced expression of cytochrome c and caspase-3 [210,215] and increased expression of BCL-2 and hypoxia-inducible factor 1-alpha (HIF1α) [210]. Resveratrol did also lead to an increased expression of PCNA, VEGF [217], and superoxide dismutase (SOD) [214,217], the latter of which is involved with the regulation of oxidative stress and inflammation (reviewed by Islam et al. [218]). Inflammatory markers (TNF-α, NF-κappaB, p65, COX-2, and iNOS) were also decreased upon resveratrol supplementation [214,215], as were oxidative damage markers (nitrotyrosine (NTY), 4-hydroxynonenal (4-HNE)) [214], all leading to an improvement in ovarian physiology and folliculogenesis (Table 5). 

Two studies also reported improvement in ovarian hyperstimulation in obesity-related infertility, diet-induced obesity [209,213], and endometritis [216]. In obesity-related infertility, decreased levels of inflammatory markers, as well as insulin resistance, abnormal lipid distribution, and oxidative stress, were observed, including decreased cytokines in the endometritis mouse model. 

#### 4.4.2. Human Studies

Two clinical studies related to the impact of resveratrol on the assisted reproduction outcome were performed (Table 6) [219,220]. The studies reported contradictory results. The Japanese study reported a negative effect of resveratrol on the clinical pregnancy rate and miscarriage [119], while no differences were observed in the Italian study [220]. The Italian study even reported a positive effect on oocyte and embryo quality and number. 

The Japanese study [219] reported a negative effect of resveratrol in women during the luteal phase and pregnancy, leading to lower implantation and higher miscarriage rates. In the study, they suggest that this could be due to the suppression of decidua senescence and the anti-inflammatory properties of resveratrol, with inflammation in early pregnancy being of great importance for successful implantation [221]. 

Despite the contrary results, a comment [222] to the Japanese study referred that it is still too early to dismiss resveratrol supplementation in embryo transfer cycles as the number of study participants was uneven and more cycles were performed in the control group (n = 7073) compared with the resveratrol group (n = 204). The average age of women in the resveratrol group was also significantly different, presenting lower AMH levels. Additionally, no information was available on the chromosomal status of the miscarried embryos [219]. The comment also suggested that resveratrol supplementation may be more beneficial to specific populations in embryo transfer cycles, like endometriosis and PCOS patients [222], which can affect pregnancy outcomes and for which the beneficial effects of resveratrol are more evident (Table 2 and Table 4). Still, caution is needed regarding the effect of resveratrol on women in the luteal phase and women undergoing pregnancy.

Due to the limited number of studies regarding the impact of resveratrol on in vitro fertilization outcomes, the beneficial evidence on assisted reproduction outcomes is, however, still limited. More randomized, preferably double-blind, placebo-controlled experimental studies are needed to be performed to determine the supplementation protocol and safety of resveratrol supplementation in women undergoing IVF and potential pregnancy.

## 5. Discussion

**Figure 2:**
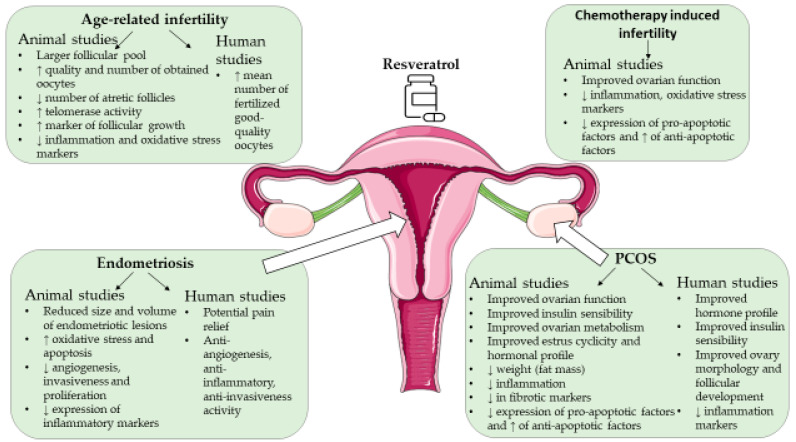
Summary of the potential beneficial effects of resveratrol on female infertility. The figure was drawn using pictures from Servier Medical Art licensed under a Creative Commons Attribution 4.0 Unported License https://creativecommons.org/licenses/by/4.0/deed.en (accessed on 17 March 2024).

In this review, numerous beneficial effects of resveratrol were reported regarding female infertility (Figure 2). Based on animal and human studies, resveratrol presented beneficial effects on follicular pool and oocyte quality in age-related infertility due to its effect on the mitochondrial membrane potential, ATP, oxidative stress levels, and reduced inflammation [122,125,126]. The effect of resveratrol on the improvement of age-related infertility could be due to resveratrol affecting numerous molecular factors, including SIRT1, p53 involved in apoptosis regulation, NF-κappaB involved with inflammation, the Akt/mTOR pathway associated with the mitochondrial function, and Nrf2 involved with the expression of antioxidant enzymes, like SOD [121]. 

Animal and human studies have also reported the potential of resveratrol in relieving PCOS symptoms such as the reduction of androgens, inflammation, fibrosis, and potential metabolic and fertility improvement of ovary and oocyte morphology and cycle regulation (references are summarized in Table 1 and Table 2). On the molecular scale resveratrol was reported to decrease pro-apoptotic factors, like Bax, decreas the expression of fibrotic factors and inflammatory markers, and increas expression of anti-apoptotic factors (BCL-2), SIRT2, and glycolysis genes. 

In female patients suffering from endometriosis, resveratrol showed potential in reducing the invasiveness of endometriosis due to reduced expression of MMP-2 and MMP-9. It also indicates anti-inflammatory and anti-vascular properties due to the reduced expression of TNF-α and VEGF, modulating angiogenesis and inflammation in endometriosis (references are summarized in Table 4). The animal models also showed the effect of resveratrol on endometriotic lesion reduction and confirmed its effect on the reduction of angiogenesis, proliferation, and invasiveness, involving molecular factors like p53, BCL-2, MMP-2 and -9, VEGF, IL-6, IL-8, TNF-α, etc. (references are summarized in Table 3). 

As resveratrol improved the chemotherapy-induced ovarian toxicity phenotype in animal models (references are summarized in Table 5), its protective effect could be of great promise for cancer patients as its use could relieve the effects of chemotherapy drugs and help preserve fertility.

Even though some randomized, double-blind studies were performed, convincing evidence is still lacking, as not all studies have managed to prove the beneficial effect of resveratrol. Nevertheless, the data on the effect of resveratrol on pregnancy and miscarriage rate were contradictory, with one study reporting negative effects and other non-significant effects on pregnancy and miscarriage rate (references are summarized in Table 6). The determination of the potential effect of resveratrol on female infertility is difficult due to the differential action of resveratrol regarding the cell type and used concentration, as also observed in female infertility, with resveratrol promoting the apoptosis in endometriotic tissue [170] while decreasing it in PCOS ovaries [148] and in ovaries being damaged due to exposure to toxic components [210,217]. Caution is also needed while choosing the preferable dose, as lower doses of resveratrol were reported as anti-apoptotic and higher as pro-apoptotic due to the effect on SIRT1 and p53 [87]. Differences were also observed regarding the cell type, with endometriotic cells being more prone to apoptosis at higher concentrations of resveratrol (>100 µM) than endometrial [197].

Regarding the study design, a large variety in the dose and duration of resveratrol treatment was observed, with the dose for animal models ranging from 1 to 400 mg/kg/day; for humans, the dose was from 30 mg a day to 1500 mg a day, and the duration was from just a few weeks to a few months. With various promising results from animal models (references are summarized in Table 1, Table 3 and Table 5), future studies should be performed to assess if the results from the animal models could be proven true in humans. More studies testing different concentrations of resveratrol are needed to explore the differential molecular mechanisms of resveratrol action based on studied cells. Regardless, resveratrol shows therapeutic potential in female infertility-related conditions, but more studies are needed to assess its safety in humans, especially for women undergoing embryo transfer and pregnancy, as the current knowledge behind its action at the molecular level is still insufficient. 

## 6. Conclusions

In recent years, resveratrol is gaining popularity, preferably due to its anti-aging, anti-cancer, and anti-inflammatory effects. The molecular mechanisms of its action are complex as it targets different molecular factors involved in various signal pathways. Numerous beneficial effects were also reported regarding female infertility, especially age-related infertility, PCOS, endometriosis, and toxicity-induced ovarian damage. Even though the results from animal models are promising, the lack of randomized, double-blind placebo-controlled studies are lacking, hindering its potential application. Caution is still needed due to the pro-apoptotic effect of higher doses and the large number of its possible molecular targets. Regardless, resveratrol shows therapeutic potential in female infertility-related conditions.

## Figures and Tables

**Table 1:** Summary of animal studies regarding resveratrol supplementation and PCOS.

Animal Model	Number of Samples	Dosage and Duration	Results	Study
Rat	Control + vehicle n = 10 Control + resveratrol n = 10 PCOS + vehicle n = 10 PCOS- + resveratrol n = 9 PCOS + exercise n = 9–10	400 mg/kg 5 days a week for 4 weeks and 7 days of the final treatment week	↓ smaller adipocytes, ↑ estrogen-related receptor α gene expression in subcutaneous fat, and improved estrus cyclicity after resveratrol and exercise No differences in body weight, Insulin sensitivity, expression of adiponectin, Fndc5, Foxo1, Nrf1, Pparg, Ppargc1a, Sirt1, CYP17a1, and Hsd3b1	[136]
Rat	Control n = 7 PCOS + saline n = 7 PCSO + Resveratrol n = 7	10 mg/kg daily for 4 weeks	↓ number of antral follicles and ↓ concentration of plasma insulin-like growth factor 1 (IGF-1) and anti-Mullerian hormone (AMH) Significantly ↓ superoxide dismutase (SOD) activity and ↑ glutathione peroxidase levels	[137]
Rat	Control n = 9 PCOS n = 9 PCOS + resveratrol n = 9 PCOS + resveratrol solvent n = 9 PCOS + metformin n = 9 PCOS + metformin solvent n = 9 PCOS + metformin + resveratrol n = 9	20 mg/kg daily for 28 days; 20 mg/kg resveratrol + 300 mg metformin daily for 28 days	↓ levels of testosterone, TNF-α, LH, LH/FSH, AMH, and malondialdehyde (MDA); a lipid peroxidation marker ↑ SIRT1 immunoreactivity Combined treatment ↓ the body and ovary weight Single or combined treatment ↓ the elevated number of secondary and atretic follicles, ↑ number of primordial and Graafian follicles, and Corpus luteum	[138]
Rat	Control n = 5 PCOS + saline n = 5 PCSOS+ resveratrol n = 5	10 mg/kg daily for 28 days	↓ HOMA-IR level and fasting serum glucose ↑ serum total antioxidant capacity ↓ serum MDA concentrations insulin levels were not statistically different	[139]
Rat	Control n = 5 PCOS + saline n = 5 PCSOS + resveratrol n = 5	10 mg/kg daily for 28 days	↓ expression of subcutaneous adipose tissue Tnf-α mRNA ↓ expression of visceral adipose tissue Tnf- α and Il-6 mRNAs	[140]
Rat	Control n = 7 PCOS n = 7 PCOS + resveratrol n = 7	100 mg/kg daily for 35 days	↓ in body weight ↓ in androgen-induced thick fibrotic capsules, the number of multiple immature follicles, and ovarian interstitial fibrosis ↑ number of luteal cells and antral follicles and serum and ovarian SOD levels ↓ ovarian oxidative stress and serum and ovary MDA levels ↓ expression of P-p66Shc, TGF-β, β-catenin, α-SMA protein, CTGF, collagen IV, and collagen IA1 ↑ expression of SIRT1 protein ↓ phosphorylation of p66Shc	[141]
Rat	Control n = 10 Vehicle n = 10 PCOS n = 10 PCOS + metformin n = 10 PCOS + resveratrol n = 10 PCOS + barberry n = 10 PCOS + barberry + resveratrol n = 10	20 mg/kg daily for 42 days; 20 mg/kg resveratrol + 3 gr/kg barberry daily for 42 days	↓ concentration of low-density lipoprotein (LDL), triglycerides, ovarian weight, MDA, TNF-α, insulin resistance, number of atretic and cystic follicles after single or combinatory treatment (alone or in combination with barberry) ↑ total antioxidant activity, levels of high-density lipoprotein (HDL), and superoxide dismutase (groups 4–7) ↓ number of cystic follicles (resveratrol, barberry, and combination group) No significant differences between serum glucose levels	[142]
Rat	Control n = 8 PCOS n = 8 PCOS + resveratrol n = 8	20 mg/kg daily for 30 days	↓ weight and concentration of serum FSH, LH, and testosterone Restoration of the estrous cycle and improved follicular development (↑ number of follicles and corpus luteum; ↓ cyst-expanded antral follicles) ↑ number of granular cells and thickness of granular layer ↑ lactate, ATP, dihydroxyacetone, beta-D-fructose 6-phosphate, and ↓ pyruvate. ↑ in glycolysis-involved genes (LDHA, HK2, PKM2, SIRT2) after resveratrol treatment	[143]
Rat	Control n = 10 PCOS n = 10 PCOS + resveratrol (40 mg/kg) n = 10 PCOS + resveratrol (80 mg/kg) n = 10 PCOS + resveratrol (160 mg/kg) n = 10	40, 80, or 160 mg/kg daily for 30 days	Normalization of plasma adiponectin and estradiol levels (↑ levels) restoration of normal ovarian morphology (↑ numbers of granule cell layers and the presence of oocytes within follicles) restoration of aromatase and nesfatin-1 expression (↑ expression)	[144]
Rat	Control n = 4 PCOS + saline n = 4 PCOS + resveratrol (20 mg/kg) n = 6 PCOS + resveratrol (30 mg/kg) n = 8	20 mg/kg and 30 mg/kg daily for 30 days	Improved ovarian tissue morphology (↑ number of granulosa cells and oocytes) ↓ weight and positive regulation of the estrous cycle ↓ cystic changes in ovary	[145]
Rat	Control n = 13 PCOS n = 18 PCOS + resveratrol n = 14 Resveratrol n = 12	20 mg/kg daily for 28 days	Improved estrus cycles and hormonal profile Improved ovarian function, ↓ AMH concentration to a normal level Protective effect on the primordial follicle pool	[146]
Rat	Control n = 8 PCOS n = 8 PCOS + resveratrol n = 8	20 mg/kg daily for 30 days	↑ increased ovarian insulin sensibility (↓ blood glucose, serum insulin, and Homeostatic Model Assessment of Insulin Resistance (HOMA-IR)) ↑ IGF1R and ↓ IGF1 of mRNA and protein levels ↑ expression of glycolytic genes (HK2, LDHA, PKM2), confirmed on protein level ↑ expression of SIRT2 (mRNA and protein)	[147]
Rat	Control n = 6 PCOS n = 6 PCOS + resveratrol n = 6	20 mg/kg/ daily for 21 days	Improved estrous cycle and granular cell layer Reversion of decreased proliferation and increased apoptosis of granulosa cells ↑ expression of LDHA, PKM2, and SIRT1 in ovarian tissue ↓ body weight ↓ HOMA-IR level ↓ testosterone Restored PCNA expression ↓ expression of Bax, caspase-3, apaf1, and cytochrome C; ↑ expression of Bcl-2 (↓ apoptosis) Restored AMP/ATP ratio	[148]

The arrows represent upregulation (↑) or downregulation (↓) of molecular factors.

**Table 2:** Summary of human studies regarding resveratrol supplementation and PCOS.

Study Type	Number of Analyzed Patients	Dosage and Duration	Nationality	Age	Results	Study
Randomized, double-blind, placebo-controlled trial	PCOS + placebo n = 15 PCOS + resveratrol n = 15	1500 mg daily for 3 months	Poland	Placebo = 26.8 ± 1.5 Resveratrol = 26.8 ± 1.1	Significant ↓ in dehydroepiandrosterone sulfate, total testosterone, fasting insulin, and ↑ Insulin Sensitivity Index. No significant changes in BMI, ovarian volume, gonadotropins, inflammation, or lipid profile	[153]
Randomized, triple-blind, placebo-controlled trial	PCOS + placebo n = 31 PCOS + resveratrol n = 30	800 mg daily for 40 days	Iran	Placebo = 30.84 ± 3.30 Resveratrol = 29.30 ± 4.44	Mean difference in LH, TSH, FSH, and testosterone (LH and testosterone ↓, FSH and TSH ↑) ↑ rate of high-quality oocytes and embryos ↓ expression of VEGF and HIF1 (pathologic angiogenesis) No significant differences between AMH levels, fertility, fertilization and cleavage rate, and oocyte maturation between groups	[154]
Randomized, double-blind, placebo-controlled trial	PCOS + placebo n = 20 PCOS + resveratrol n = 20	800 mg daily for 40 days	Iran	Placebo = 30.35 ± 4.00 Resveratrol = 29.55 ± 3.28	↓ in inflammatory markers, like IL-18, CRP, and borderline TNF-α in comparison to the placebo group. IL-6 and IL-1β levels also ↓ after resveratrol treatment, but not statistically significant ↓ Level of NF-κappaB differences in gene expression (↑ expression of ATF4 and ATF6, involved with unfolding protein response due to ER stress, ↓ expression of CHOP, GRP78, and XBP1)	[155]
Randomized, double-blind, placebo-controlled trial	PCOS + placebo n = 39 PCOS + resveratrol n = 39	1000 mg of resveratrol daily for 3 months	Iran	Placebo = 27.87 ± 6.24 Resveratrol = 26.33 ± 5.62	↑ menstruation regularities, ↓ hair loss No significant differences in ovarian and adrenal androgens, sex hormone binding globulin (SHBG) levels, free androgen index (FAI), lipid, and glycoinsulinemic profile	[156]
Randomized, double-blind, placebo-controlled trial	PCOS + placebo n = 17 PCOS + resveratrol n = 19	1000 mg daily for 3 months	Iran	Placebo = 27.30 ± 5.22 Resveratrol = 29.79 ± 4.61	Improvement of the polycystic ovarian morphology ↓ ovarian volume No significant differences between the number of follicle count per ovary (FNPO), stromal area (SA), ovarian echogenicity, and distribution of follicles	[157]

The arrows represent upregulation (↑) or downregulation (↓) of molecular factors.

**Table 3:** Summary of animal studies regarding resveratrol supplementation and endometriosis.

Animal Model	Number of Samples	Dosage and Duration	Results	Study
Mouse	Control n = 16 Resveratrol n= 20	6 mg/mouse for 10–12 or 18–20 days	↓ in the number, size, and total volume of endometriotic implants	[169]
Mouse	Control n = 8 Resveratrol (10 mg/kg) n = 10 Resveratrol (25 mg/kg) n = 10	10 mg/kg or 25 mg/kg daily for 4 weeks	↓ in the mean number and volume of endometriotic lesions ↓ vascular density and cell proliferation and ↑ apoptosis in endometriotic lesions	[170]
Mouse	Control n = 10 Resveratrol n = 10	40 mg/kg daily for 4 weeks	↓ microvessel density (angiogenesis inhibition in endometriotic lesions; ↓ proliferation of endothelial cells) ↓ growth and size of endometriotic lesions (↓ proliferation of stromal and glandular cells)	[171]
Rat	Control n = 6 Resveratrol n = 6	10 mg/kg daily for 14 days	↓ in the endometriotic implant size ↓ levels of vascular endothelial growth factor (VEGF) in the plasma and peritoneal fluid ↓ levels of monocyte chemotactic protein 1 (MCP-1) in the peritoneal fluid ↑ suppression of VEGF expression in the endometriotic tissue Histological changes (↓ vascularization)	[172]
Mouse	Estradiol n = 4 Estradiol + progesterone n = 4 Estradiol + resveratrol (6 mg/kg) n = 4 Estradiol + resveratrol (30 mg/kg) n = 4 Estradiol + resveratrol (60 mg/kg) n = 4	6, 30, or 60 mg daily for 30 days	↓ expression of ESR1 and ↓ proliferative activity (60 mg/kg)	[173]
Rat	Control n = 8 Resveratrol (1 mg/kg) n = 8 Resveratrol (10 mg/kg) n = 8	1 mg/kg or 10 mg/kg daily for 7 days	↓ endometriotic implant volume ↑ (dose-dependent) activity of superoxide dismutase and glutathione peroxidase in serum and tissue in resveratrol groups ↑ MDA levels and catalase levels in serum and tissue in 10 mg/kg resveratrol group ↓ proliferating cell nuclear antigen expression and histological scores in resveratrol groups	[174]
Rat	Control n = 7 Leuprolide acetate n = 8 Resveratrol n =7	60 mg/kg daily for 21 days	↓ mean size of endometriotic implants and histopathological score ↓ VEGF-staining scores and peritoneal fluid levels of VEGF and MCP-1 ↓ serum VEGF and MCP-1	[175]
Rat	Control n = 8 Resveratrol n = 9 Leuprolide acetate n = 8 Resveratrol + leuprolide acetate n = 8	30 mg/kg daily for 14 days	↓ endometriotic implant volume ↓ histopathological score ↓ levels of IL-6, IL-8, and TNF-α in plasma and peritoneal fluid ↓ expression of MMP-2, MMP-9 and VEGF	[176]
Mouse	Control (PBS) n = 6 Blank n = 6 Resveratrol n = 6	25 mg/kg daily for 4 weeks	↓ growth of ectopic endometriotic lesions ↓ expression of MTA1 and ZEB2 (involved with epithelial-mesenchymal transition)	[177]
Rat	Endometriosis control n = 6 Atorvastatin n = 6 Resveratrol n = 6 Resveratrol + atorvastatin n = 6	40 mg kg daily for 28 days	↓ in ectopic endometrial tissue size and neovasculature ↓ expression of GLUT-1, GLUT-3, MCT-1, and MCT-4 ↓ distribution of GLUT-1+, GLUT-3+, and MCT-4+ cells per mm2 of tissue	[178]
Rat	Control n = 10 Endometriosis n = 10 Resveratrol (15 mg/kg) n = 10 Resveratrol (45 mg/kg) n = 10	15 mg/kg and 45 mg/kg daily for 28 days	↓ endometriotic lesion size in both groups Better histology (↓ glandular tubes and endometrial epithelial thickness at histology) in both groups ↓ cholesterol, HDL, and LDL levels in the medium dose group (15 mg/kg) ↓ cholesterol and HDL in high-dose group (45 mg/kg) ↓ expression of MMP-2, VEGF, and BCL-2 Induction of PPARα	[179]
Rat	Control n = 10 Endometriosis n = 10 Resveratrol (15 mg/kg) n = 10 Resveratrol (45 mg/kg) n = 10)	15 mg/kg and 45 mg/kg daily for 28 days	↓ in the volume of endometriotic lesions and adhesion 2123 differentially expressed genes (↑ expression of genes involved with blood vessel morphogenesis, transmembrane transport, ↓ expression of genes involved with immunity activation and regulation) ↓ in INF-γ, IL-6 TNF-α, and ↑ of IL10 Induction of PPARγ Changes in glucose tolerance, adipocyte size, and macrophage polarization	[180]
Mouse	/	25 mg/kg daily for 14 days	↓ volume of the endometriotic lesions ↓ cell density of the endometriosis cyst tissue ↓ serum GSH and ↑ MDA levels ↑ ROS ↑ expression of p53 and ↓ expression of miR-21-3p and SLC7A11 ↑ expression of Chac1 and Ptgs2 Promotion of ferroptosis	[181]

The arrows represent upregulation (↑) or downregulation (↓) of molecular factors.

**Table 4:** Summary of human studies regarding resveratrol supplementation and endometriosis.

Study Type	Number of Analyzed Patients	Dosage and Duration	Nationality	Age	Results	Study
Experimental	Endometriosis: Office-based study n = 12 Immunohistochemistry study n = 42	30 mg daily for 2 months + oral contraceptives	Brazil	Office-based study = 30 ± 5 Immunohistochemistry study = 31 ± 4	↓ endometriosis-associated dysmenorrhea, potentiation of the effect of oral contraceptives ↓ aromatase and cyclooxygenase-2 expression in the endometrium	[198]
Randomized double-blind, placebo-controlled trial	Resveratrol n = 22 Placebo n = 22	40 mg daily for 42 days + monophasic oral contraceptives	Brazil	Placebo = 32.4 ± 7 Resveratrol 35.4 ± 7.1	No significant differences between pain relief in comparison to placebo	[199]
Randomized double-blind, placebo-controlled trial	Resveratrol n = 17 Control n = 17	400 mg twice daily for 12–14 weeks	Iran	Control = 31.32 ± 1.71 Resveratrol 30.19 ± 2.40	↓ in mRNA and protein levels of MMP-2 and MMP-9 in the endometrium, serum, and endometrial fluid	[200]
Randomized double-blind, placebo-controlled trial	Resveratrol n = 17 Control n = 17	400 mg daily for 12–14 weeks	Iran	Control = 31.32 ± 1.71 Resveratrol 30.19 ± 2.40	↓ gene and protein level of VEGF and TNF-α in the eutopic endometrium	[201]

The arrows represent upregulation (↑) or downregulation (↓) of molecular factors.

**Table 5:** Summary of animal studies regarding resveratrol supplementation and female infertility due to other causes.

Animal Model	Number of Samples	Dosage and Duration	Results	Study
Mouse	Placebo n = 8 Resveratrol n = 8 Obesity placebo n = 8 Obesity resveratrol n = 8	3.75 mg/kg daily for 20 days	Ovarian hyperstimulation in obesity-related infertility: ↓ levels of plasma insulin and testosterone in obese mice Improvement of Homeostatic Index of Insulin Resistance in obese mice Normalization of IL-6 and TNF-α levels in obese mice ↓ number of primary, growing, preovulatory, and atretic follicles in obese mice ↑ number of retrieved oocytes in non-obese mice	[209]
Rat	Control n = 10 Hexavalent chromium n = 10 Hexavalent chromium + Resveratrol n = 10	10 mg/kg daily for 21 days	Chromium-toxicity: Protective effect against chromium toxicity in the ovarium ↑ expression of cell survival proteins: Bcl-2, HIF1α (↓ apoptosis) ↓ expression of cytochrome C and cleaved caspase-3 ↓ in oocyte and granulosa cell apoptosis ↓ oxidative stress (↑ antioxidants) Restoration of estradiol levels (↑)	[210]
Mouse	Control n = 60 Mancozed n = 60 Mancozed + resveratrol (100 mg/L) n = 60 Mancozed + resveratrol (200 mg/L) n = 60	100 mg/L or 200 mg/L daily for 4 weeks	Mancozeb (fungicide) toxicity: improvement of mancozeb-induced decrease in fertility, ovary weight, and primary follicles ↑ litter size and weight ↓ ROS, apoptosis Improved oocyte quality and development potential Improvement of abnormal epigenetic modification	[211]
Rat	Control n = 7 Cisplatin + resveratrol (5 mg/kg) n = 7 Cisplatin + resveratrol (25 mg/kg) n =7 Cisplatin + saline n = 7	5 mg/kg + cisplatin or 25 mg/kg + cisplatin daily for 21 days	Cisplatin (chemotherapeutic) toxicity: prevention of cisplatin-induced ovarian damage ↑ numbers of primary and primordial follicles (5 mg/kg) no significant differences in AMH levels	[212]
Mouse	Control n = 15 High fat diet + resveratrol = 15 High-fat diet + saline = 15	10 mg/kg/daily for 3 weeks	Diet-induced obesity: Protective effect on ovary ↓ in the negative effect of diet-associated obesity on oocyte quality ↓ number of destroyed follicles Restoration of oocyte zona pellucida (proper hardness) ↓ in the abnormal lipid distribution ↓ oocyte ROS levels Protective effect on mitochondrial damage ↓ in obesity-related abnormal spindle morphology and chromosomal abnormalities	[213]
Mouse	Conntrol n = 15 Cyclophosphamide + busulfan n =15 Cyclophosphamid + busulfan + resveratrol (30 mg/kg) n = 15 Cyclophosphamid + busulfan + resveratrol (100 mg/kg) n = 15	30 or 100 mg/kg/daily for 2 weeks.	Chemotherapy toxicity: Improvement of chemotherapy-induced ovarian aging (30 mg dose) ↓ oogonial stem cell loss (30 mg dose) ↑ expression of c-KIT, Oct4, Sox2, Nanog, Gdf9 and Ddx4 (30 mg dose) ↑ SOD2 and ↓ oxidative damage markers (NTY and 4-HNE) (30 mg dose) ↑ expression of SIRT1, FOXO1, and ↓ expression of NF-κappaB (30 and 100 mg dose) Improvement of oogonial stem cell viability at low doses (2 and 5 μM) at decreased at low doses (50, 100, and 200 μM) ↑ expression of SOD2 and Nrf2 and ↓ expression of caspase-3 and bax ↓ oogonial stem cell apoptosis	[214]
Rat	Control n = 15 Cisplatin n = 15 Resveratrol n = 15 Resveratrol + cisplatin n = 15	10 mg/kg daily for 17 days	Cisplatin (chemotherapeutic) toxicity: Improvement of follicle morphology ↑ levels AMH (decreased due to cisplatin) ↓ inflammatory markers (TNF-α, NF-κappaB, p65, COX-2, and iNOS) ↓ expression of cytochrome c and caspase-3 ↓ expression of poly(ADP-ribose) polymerase (PARP-1)	[215]
Rat	Control n = 6 Endometritis n = 6 Endometritis + marbofloxacin + PGF2α n = 6 Endometritis + marbofloxacin = 6 Endometritis + marbofloxacin + resveratrol n = 6 Resveratrol n = 6	30 mg/kg for 14 days	Endometritis: Better healing (macroscopic) of uterine and ovarian tissue ↓ serum cytokine levels CINC-3, CNTF, LIX, IL-4, IL-6, and CINC-1/CXCL-1 (resveratrol alone or in combination). Not significant: IL-10, CINC-2/CXCL-3, and TNF-α ↑ total antioxidant status ↓ oxidative stress index	[216]
Mouse	Control n = 9 Doxorubicin n = 9 Doxorubicin + resveratrol (7 mg/kg) n = 9 Doxorubicin + resveratrol (15 mg/kg) n = 9	7 and 15 mg/kg seven doses, one every 48 h	Doxorubicin (chemotherapeutic) toxicity: ↑ number of primary and antral follicles, preservation of primordial follicle number ↓ number of atretic follicles ↑ number of AMH-positive follicles ↓ DNA damage and apoptosis in preantral and early antral follicles ↑ proliferation index in follicular cells ↑ PCNA expression ↑ VEGF expression Restored architecture of the uterine tissue ↑ SOD expression (antioxidant maintenance)	[217]

The arrows represent upregulation (↑) or downregulation (↓) of molecular factors.

**Table 6:** Summary of human studies regarding resveratrol supplementation and female infertility due to other causes.

Study Type	Number of Analyzed Patients	Dosage and Duration	Nationality	Age	Results	Study
Cross-sectional retrospective study	Control n = 2958 Resveratrol n = 102	200 mg daily Continuously (IVF embryo transfer cycles)	Japan	Control = 37.0 ± 3.78 Resveratrol 39.1 ± 3.01	↓ clinical pregnancy rate ↑ risk of miscarriage	[219]
Randomized, single-blind, controlled experimental study	Control n = 50 Resveratrol n = 40	150 mg daily for 3 months + folic acid (400 mcg), vitamin D (25 mcg), vitamin B12 (2.5 mcg), and vitamin B6 (1.4 mg)	Italy	Control = 36.6 ± 0.6 Resveratrol 36.1 ± 0.6	↑ number of oocytes and MII oocytes ↑ fertilization rate ↑ number of cleavage embryos/blastocytes and cryopreserved embryos per patient No significant differences between pregnancy rates and miscarriage and live birth rates	[220]

The arrows represent upregulation (↑) or downregulation (↓) of molecular factors.

## Data Availability

Not applicable.
